# Safety and Feasibility of Fan Therapy for Dyspnea: A Scoping Review

**DOI:** 10.7759/cureus.43668

**Published:** 2023-08-17

**Authors:** Tomoo Sato, Shunsuke Taito, Yuki Nakashima, Kotomi Sakai, Jun Kako

**Affiliations:** 1 Acute Care Nursing Division, Kobe City College of Nursing, Kobe, JPN; 2 Division of Rehabilitation, Hiroshima University Hospital, Hiroshima, JPN; 3 Department of Systematic Reviewers, Scientific Research Works Peer Support Group (SRWS-PSG), Osaka, JPN; 4 Comprehensive Unit for Health Economic Evidence Review and Decision Support (CHEERS) Research Organization of Science and Technology, Ritsumeikan University, Kyoto, JPN; 5 Department of Nursing, Mie University, Mie, JPN

**Keywords:** icu( intensive care unit ), intensive care, respiratory care, intensive respiratory care, critical ill, fan therapy, dyspnea

## Abstract

Fan therapy is a non-pharmacological approach useful in terminally ill patients that relieves dyspnea by directing a fan to blow air on one side of the patient’s face. To date, there has been no systematic review of fan therapy for critically ill patients in the intensive care unit. This scoping review aimed to provide a comprehensive overview of fan therapy studies published to date, clarify the therapeutic intervention methods of fan therapy, evaluate its safety according to existing literature, and explore its potential use in critically ill patients. A scoping review was conducted using the Joanna Briggs Institute methodology. This scoping review follows the Preferred Reporting Items for Systematic Reviews and Meta-Analyses extension of the scoping reviews statement. All published studies conducted on patients who received fan therapy regardless of age, disease, setting, phase, country, or follow-up duration were included. The data sources included Medical Literature Analysis and Retrieval System Online, Embase, Cochrane Central Register of Controlled Trials, and Cumulative Index to Nursing and Allied Literature databases. Of the 685 studies obtained, 15 were included, comprising patients with terminal cancer and chronic lung diseases. The most common intervention was a single five-minute intervention for dyspnea at rest. The studies on patients receiving oxygen therapy did not report adverse events or worsening of blood pressure, pulse rate, respiratory rate, or SpO_2_ levels. However, there are no studies in the literature on the use of fan therapy for critically ill patients. Nevertheless, previous studies suggest that fan therapy is safe.

## Introduction and background

Dyspnea is “a subjective experience of breathing discomfort that consists of qualitatively distinct sensations that vary in intensity” [1]. Dyspnea is believed to occur when there is a mismatch between motor commands from the respiratory center to the respiratory muscles and afferent information from nerves and other receptors. Dyspnea in the intensive care unit (ICU) is one of the most distressing experiences [2] and is associated with patient death [3]. It has also been suggested to play an important role in the development of ICU-related posttraumatic stress syndrome [4,5]. In a prospective observational study of critically ill patients in the ICU, the prevalence of dyspnea ranged from 34% to 63% [6-8]. In critically ill patients, treating dyspnea is essential to resolve the underlying cause; however, a resolution is often impossible or does not provide adequate symptom relief. In such cases, palliative interventions that target the symptom complex may effectively reduce symptom burden and distress. Fan therapy is one such intervention.

**Figure 1:**
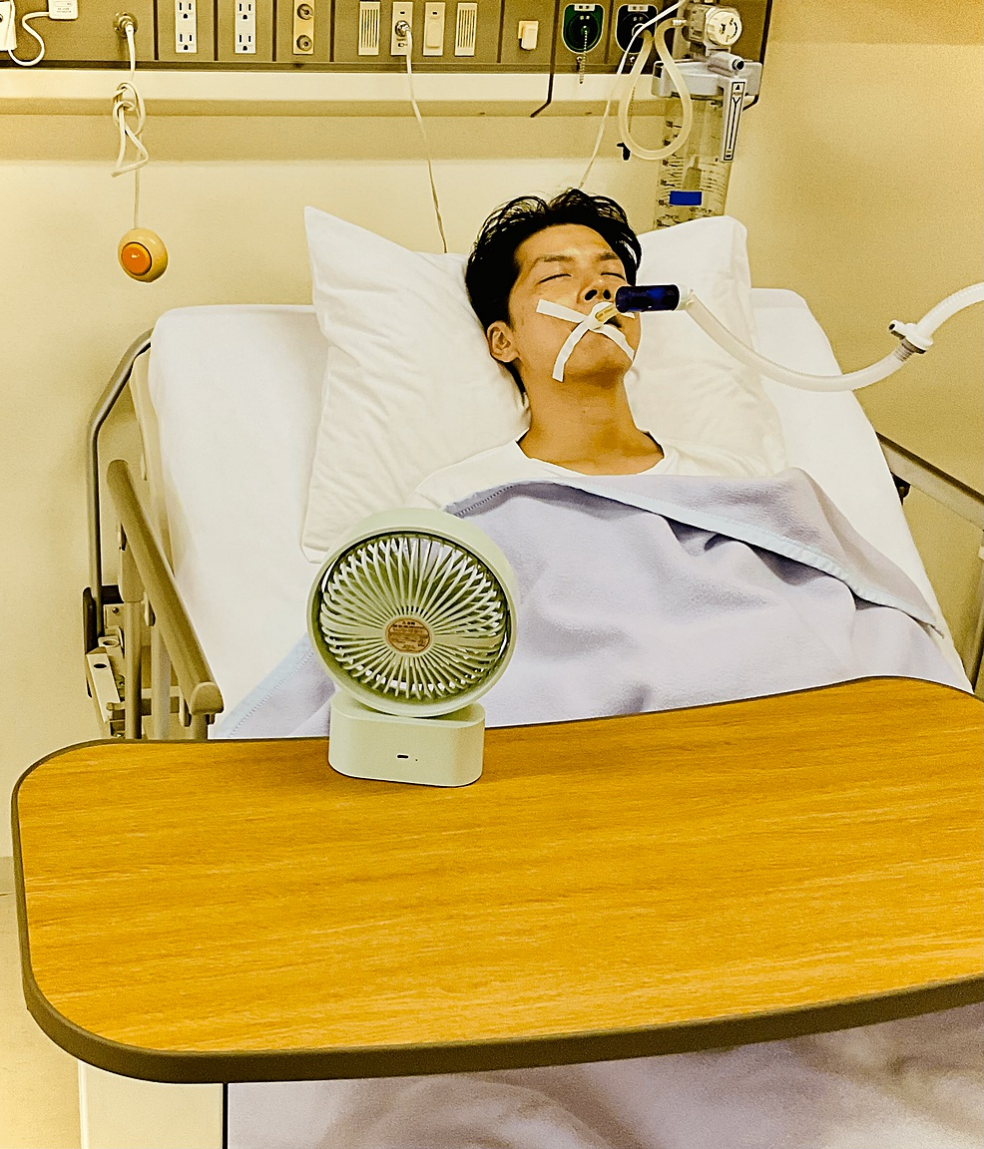
Fan therapy Fan therapy consists of holding the fan at a convenient distance from the face and directing the wind to the areas innervated by the second and third branches of the trigeminal nerve.

Fan therapy is a non-pharmacological approach that relieves dyspnea by directing a fan to blow air on one side of the patient’s face. Figure 1 illustrates the fan therapy. However, the mechanisms underlying its effects remain unclear. It has been hypothesized that direct stimulation of the face (around the second/third trigeminal nerve branches), nasal mucosa, pharynx, or changes in facial temperature due to cooling, may affect ventilation patterns [9-11]. Several clinical studies have recommended fan therapy for dyspnea, and there is emerging evidence of its efficacy [12-14]. Therefore, fan therapy should be considered at the end of life and in critically ill patients with dyspnea in the ICU.

The objective of this scoping review was to provide a comprehensive overview of published studies on fan therapy to clarify the therapeutic intervention methods of fan therapy, evaluate its safety according to existing literature, and explore its potential use in critically ill patients.

## Review

Protocol and registration

In accordance with a predefined protocol [15], we conducted a scoping review based on the following five-stage framework outlined by the Joanna Briggs Institute: identifying the research question; identifying relevant studies; study selection; data charting; and collating, summarizing, and reporting the results [16]. This scoping review follows the Preferred Reporting Items for Systematic Reviews and Meta-Analyses extension of the scoping reviews statement (Appendix 1) [17]. Full details of the methodology can be found in the scoping review protocol registered as a priori in the Open Science Framework on January 9, 2023 [15].

Eligibility criteria and search strategy

Population, concept, and context frameworks were used to define the inclusion criteria [16]. All published studies conducted on patients with fan therapy, defined as the “use of an electrical fan blowing on the patient’s face,” [18], and studies using airflow, table fan, standing fan, and handheld fan were included in this study. Studies were included regardless of age, disease, setting (e.g., hospital, home, and institutional), phase (e.g., acute phase, chronic phase, terminal phase), country, or follow-up duration of the patients. Studies that did not fit the conceptual framework of the present review were excluded. All published randomized controlled trials (RCTs); crossover, cluster randomized, quasi-randomized, and non-randomized trials; observational studies with controls; case reports; and case series were included in the analysis. Studies in any language or country were accepted, and studies with any length of follow-up were included. Conference abstracts and review articles were excluded from the analysis.

The following databases were comprehensively searched on December 28, 2022: Medical Literature Analysis and Retrieval System Online (MEDLINE), Excerpta Medica Database (Embase), Cochrane Central Register of Controlled Trials (CENTRAL), and the Cumulative Index to Nursing and Allied Literature (CINAHL). In addition, searches were conducted using the World Health Organization International Clinical Trials Registry Platform and ClinicalTrials.gov to identify ongoing clinical trials. ​​The search formulas are presented in Appendix 2. We also identified additional relevant studies by manually searching the reference lists of the included studies and relevant reviews (based on citation information from the Web of Science).

Study selection

Two reviewers (TS and YN) independently assessed the titles and abstracts, followed by an assessment of eligibility based on the full texts. If the relevant data were missing, the original authors were contacted. Disagreements between the two reviewers were resolved by discussion. If this failed, a third reviewer (KS) acted as an arbiter.

Data extraction and synthesis

Data extraction was conducted by a researcher (TS) using a standard data extraction form, including disease, setting, study type, number of participants, fan therapy intervention, control, outcomes, changes in respiratory rate, SpO_2_ levels, pulse rate, blood pressure before and after fan therapy, adverse events, and feasibility. Another researcher (YN) verified the data extraction process. Where necessary, the authors of the reviewed publications were contacted. We organized the extracted data described above for qualitative synthesis.

Results

Selection of Sources of Evidence

**Figure 2:**
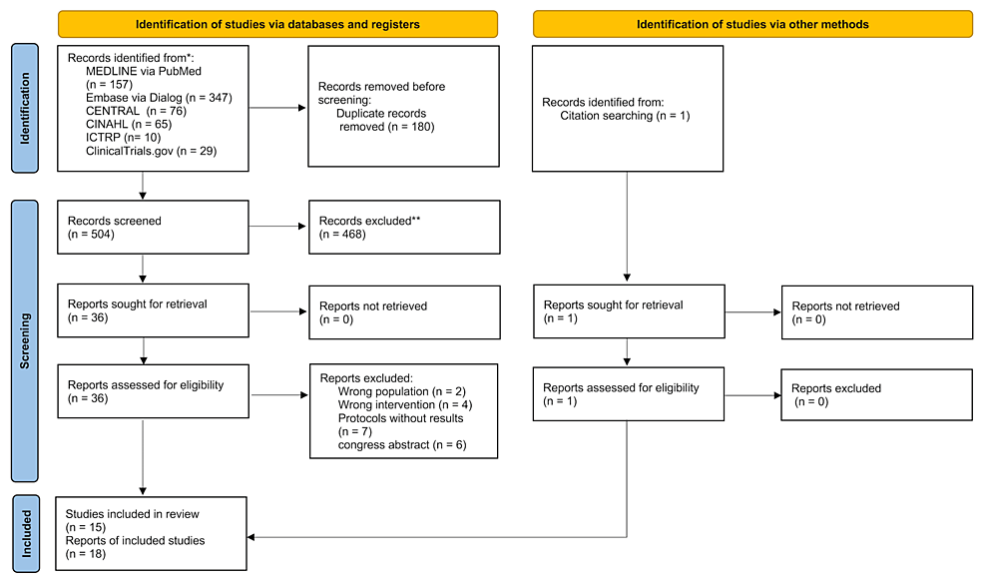
PRISMA flow diagram of the literature search results

Of the 685 articles identified, 15 studies (18 reports) were included in this scoping review (505 patients; Figure 2) [12,13,18-30].

Characteristics of Sources Evidence

The study selection process is illustrated in Figure 2. In total, 15 studies were conducted in the United Kingdom [12,18,25,27,29], Japan [20-22], Australia [23,28], Germany [19], Turkey [24], United States of America [26], Philippines [30], and China [13]. Regarding the study design, this review included five parallel RCTs [13,19,22-24], three crossover RCTs [18,25,30], one crossover non-RCT [27], two non-RCT parallels [22,26], one before-after trial [28], one observation trial [12], one case series [20], and one case report [29].

Results of Individual Sources of Evidence

Patients in 15 studies were in terminal phases of cancer [12,13,18-22,24,29,30], such as lung, pancreatic, and breast cancers, or chronic phase of chronic obstructive pulmonary disease (COPD) [12,18,19,25-28], interstitial lung disease (ILD) [24], asthma [5], or heart disease [19,20]. Studies have been conducted on fan therapy in the palliative care unit [13,20-22,24,29], ward [12,18,19,23,28,30], home [19], outpatient [12,25,27] and clinic [19,26] (Table 1). There were no intervention studies using fan therapy in the critical care phase. Although most ongoing studies awaiting classification included patients in the terminal and chronic phases, two RCTs included patients in the critical care phase admitted to the ICU [31,32]. Dyspnea at baseline in 15 of the studies was evaluated using a numerical rating scale (NRS), 5.0-6.1 [12,13,21,22,25]; visual analog scale (VAS), 31.0-48.5 [12,18,20]; Borg scale (Borg), 4 [19]; modified Borg scale (mBorg), 1.5-7.5 [24,27,30]; modified Medical Research Council dyspnea scale (mMRC), 3 [28]; and dyspnea-12, 16.1 [23]. The fan therapy interventions in the studies consisted of seven handheld fans [12,18,19,23-25,28], six standing fans [20-22,26,27,30], and two table fans [13,29], and were administered at rest [12,13,18,20-22,28,30] or during exercise (Table 1) [23,25-27]. Seven studies reported the proportion of patients on oxygen therapy [20-23,26,27,30], of which five [20-23,26] found that the proportion of patients on oxygen therapy ranged from 13 to 67%. In two studies, the proportion was 0% [27,30]. Twelve of the studies had a control group with five fan-to-leg movements [18,21,22,26,30], four had no intervention [21,23-25], one used compressed air [27], one was accompanied by caregivers [13], and one used wristbands [19]. More than half of the studies had only one five-minute intervention each [12,13,18,20-22,29,30].

**Table 1:** Fan therapy 6MWT: six-minute walk test; ECOG: Eastern Cooperative Oncology Group; ESAS-r: Edmonton Symptom Assessment System Revised; FACIT- Pal: Functional Assessment of Chronic Illness Therapy-Palliative Care; ILD: interstitial lung disease; mBorg: modified Borg scale; mMRC: modified Medical Research Council Dyspnea Scale; NR: not recorded; NRS: numerical rating scale; RCT: randomized controlled trial; VAS: visual analog scale.

Author, year, country	Study type	Phase (Setting)	Disease	Dyspnea at baseline, mean (SD)	Oxygen therapy, N (%)	Fan therapy intervention	Control	Outcome
									Booth et al. [12], 2016, United Kingdom	Observation trial	Chronic (Ward, Outpatient)	Mixed non- malignant (n=8), Cardiorespiratory disease (n=7), COPD (n=7), Cancer (n=5), Other (n=4)	VAS 48.5 (27.4), NRS 5.5 (2.2)	NR	At a comfortable distance of 15–30 cm from the face, blow air for 5 min across the areas innervated by the second/third branches of the trigeminal nerve.	No intervention	(i) Scores had returned to baseline values of dyspnea, (ii) Relief from breathlessness noted on relief score (VAS and NRS), (ii) Relief from breathlessness noted on relief score (VAS and NRS), (iv) The absolute change in scores from baseline values, (v) The correlation between the changes in VAS, NRS, and relief scores
									Wong et al. [13], 2017, China	Parallel RCT	Terminal (Palliative care unit)	Lung cancer (n=13), Other cancer (n=17)	NRS 6.1 (2.5)	NR	Patients were given fan therapy, which involved the use of a table fan to blow air to the face for 5 minutes.	Accompanied by their caregivers	(i) NRS, (ii) SpO2, (iii) Respiratory rates
Galbraith et al. [18], 2010, United Kingdom	Crossover RCT	Chronic and Terminal (Ward)	COPD (n=26), Heart disease (n=15), Lung cancer (n=11), Asthma (n=8), Bronchiectasis (n=7), Pneumonitis (n=4), Other (n= 20), Multiple diagnoses (up to 4 in any one patient) (n=26)	VAS 31 (36.3)	NR	Use a handheld fan for five minutes directed to their face.	Fan to leg	(i) VAS, (ii) SpO2, (iii) Pulse rate
Bausewein et al. [19], 2010, Germany	Parallel RCT	Chronic and Terminal (Ward, home, clinic)	COPD (n=45), Lung cancer (n=25)	Borg score 4 (1.9)	NR	A hand-held fan was directed to the area of the face in nervated by the second and third trigeminal nerve branches for two months.	Wore a wristband	(i) Status of use, (ii) Questions if it is helpful after 2 months, (iii) Change of breathlessness severity (mBorg) after 2 months, (iv) Uptake into the trial
Kako et al. [20], 2015, Japan	Case series	Terminal (Palliative care unit)	Cancer (n=9)	VAS 40.2 (11.8)	6 (67)	Participants used a standing fan to blow air by for 5 minutes across the region innervated by the second/third trigeminal nerve branches.	No intervention	(i) VAS, (ii) Satisfaction with fan therapy, (iii) Pulse rate, (iv) Respiratory rates, (v) SpO2, (vi) pain VAS
Kako et al. [21], 2018a, Japan	Non-RCT	Terminal (Palliative care unit)	Lung cancer (n=6), Other cancer (n=3)	NRS 5.9 (1.8)	6 (67)	Participants used a standing fan to blow air by for 5 minutes across the region innervated by the second/third trigeminal nerve branches.	(i) Fan to leg, (ii) No intervention	(i) Duration required for the dyspnea score to return to baseline after the intervention, (ii) Relief from breathlessness, as measured by the NRS during each intervention, (iii) Patients’ preferred type of therapy, (iv) Change in the patients’ face surface temperature
Kako et al. [22], 2018b, Japan	Parallel RCT	Terminal (Palliative care unit)	Lung cancer (n=15), Pancreas cancer (n=4), Colon/rectum cancer (n=3), Breast cancer (n=3), Head and neck cancer (n=3), Stomach cancer (n=2), Prostate cancer (n=2), Esophagus cancer (n=2), Gallbladder/bile duct cancer (n=2), Other cancer (n=4)	NRS 5.3 (1.4)	9 (45)	Fan therapy constituted of directing a standing fan to blow air for five minutes across the region innervated by the second/third trigeminal nerve branches.	Fan to leg	(i) NRS, (ii) ESAS-r (pain, tiredness, drowsiness, nausea, lack of appetite, depression, anxiety, dyspnea, and well-being), (iii) Facial surface temperature, (iv) Respiratory rate, (v) SpO2, (vi) Pulse rate
Khor et al. [23], 2021, Australia	Parallel RCT	Chronic (Ward)	Interstitial lung disease (n=30)	Dyspnea-12 16.1 (2.2)	2 (13)	The handheld fan should be about 15 cm away from the face, with the airflow directed toward the center of the face, so that it can be felt next to the nose and above the upper lip, and can be used as often as needed for two weeks, when resting after activity, or when feeling suffocated at rest.	No intervention	(i) Change in Dyspnoea-12 scores, (ii) Participants’ perspectives regarding the use of fan for managing their symptoms, (iii) HRQoL, (iv) Self-efficacy, (v) Functional performance, (vi) King's Brief Interstitial Lung Disease Questionnaire, (vii) Self-efficacy for Managing Chronic Disease 6-item Scale
Kocatepe et al. [24], 2021, Turkey	Parallel RCT	Terminal (Palliative care unit)	Lung cancer (n=96)	mBorg 6 (1.2)	NR	Use the handheld fan—held 15 cm away from the face—for five minutes three times per day (before breakfast, lunch, and dinner) for 14 days.	No intervention	(i) mBorg, (ii) Respiration rate, (iii) Oxygen saturation, (iv) Heart rate, (v) ECOG performance status scale, (vi) FACIT-Pal, (vii) Pulmonary function test , (viii) Arterial blood gas
Long et al. [25], 2021, United Kingdom	Crossover RCT	Chronic (Outpatient)	COPD (n=14)	NRS 5 (1.9)	NR	Patients use the handheld fan: to hold the fan to their face throughout both the walking test (6MWT) and during the recovery period until they reported that breathlessness returned to baseline.	No intervention	(i) NRS, (ii) Breathlessness intensity (NRS), (iii) Perceived breathing difficulty/effort (mBorg), (iv) Distance walked (m) during the 6MWT, (v) Post-exertional recovery time, (vi) Acceptability of using the fan (Likert scale questionnaire)
Marchetti et al. [26], 2015, United States of America	Non-RCT	Chronic (Clinic)	COPD (n=10)	NR	4 (40)	During the exercise in ergometry, a standing fan with a diameter of 12 cm is aimed at the patient's face to blow air.	Fan to leg	(i) Total exercise time, (ii) Less dynamic hyperinflation, (iii) Tidal volume, (iv) Respiratory rate, (v) Heart rate, (vi) Blood pressure
O'Driscoll et al. [27], 2011, United Kingdom	Crossover non-RCT	Chronic (Outpatient)	COPD (n=34)	mBorg 1.5 (1.1)	0 (0)	During a simple step test on an exercise step, a standing fan with 28 cm blades blows cold air on the face from a distance of 1 m.	(i) room air, (ii) compressed air from a face-mask	(i) mBorg, (ii) The mean number of steps climbed, (iii) SpO2, (iv) Pulse rates, (v) Post-exertional recovery time
Smith et al. [28], 2022, Australia	Before-after trial	Chronic (Ward)	COPD (n=33)	mMRC 3	NR	Participants used a hand-held fan, directed at their face for a maximum of 1 minute.	NR	(i) Perceived airflow (NRS), (ii) Pleasantness of airflow (NRS), (iii) Ease of use (NRS), (iv) Noisiness (NRS)
Sutherl et al. [29], 2022, United Kingdom	Case reports	Terminal (Palliative care unit)	Metastatic small cell lung cancer (n=1)	NR	NR	The clinical team turned on the table fan, using the oscillating motion setting, angled at 90° to her face and at 50 cm distance for 5min.	NR	The cough frequency after 5 min
Ting et al. [30], 2020, Philippines	Crossover RCT	Terminal (Ward)	Lung cancer (n=10), Breast cancer (n=8), Osteosarcoma: leg (n=4), Prostate cancer (n=4), Esophageal cancer (n=3), Head and neck cancer (n=3), Germ cell tumor (n=3), Colorectal cancer (n=2), Bladder cancer (n=2), Lymphoma cancer (n=2), Other cancer (n=7)	mBorg 7.5 (0.5)	0 (0)	Fan on face therapy involved directing a standing fan to blow air for 5 minutes across the region innervated by the second/third trigeminal nerve branches.	Fan to leg	(i) mBorg, (ii) Oxygen saturation, (iii) Respiratory rates

Measurements of SpO_2_ were performed in nine studies [12,13,20-22,24,25,27,30], respiratory rate in six studies [13,20-22,24,30], pulse rate in seven studies [12,20-22,24,25,27], and blood pressure in one study [24]. O’Driscoll et al. decreased the SpO_2_ and increased the pulse rate because they were measured before and after a simple step test [27]. This study reported no differences between the fan therapy and control groups [27]. Most studies measured SpO_2_ at rest, and no worsening of SpO_2_, respiratory rate, pulse rate, or blood pressure was reported before or after fan therapy [12,13,18,20-22,25,28,30]. Eight studies [18-22,27,28,30] reported no adverse events and seven studies [12,13,23-26,29] did not report adverse events as outcomes (Table 2).

**Table 2:** Safety and feasibility of fan therapy for patients NR: not recorded

Author, year	SpO_2_, *mean *		Respiratory Rate, *mean*		Pulse Rate, *mean *		Blood Pressure, *mean *		Adverse events,* N*	Withdrawals, *N* (*%*)	Adherence, *%*
	before	after	before	after	before	after	before	after			
Booth et al. [12], 2016	92.1	NR	NR	NR	84.2	NR	NR	NR	NR	0 (0)	100
Wong et al. [13], 2017	93.4	93.4	21.5	20.7	NR	NR	NR	NR	NR	0 (0)	100
Galbraith et al. [18], 2010	NR	NR	NR	NR	NR	NR	NR	NR	0	1 (2)	100
Bausewein et al. [19], 2010	NR	NR	NR	NR	NR	NR	NR	NR	0	14 (37)	48
Kako et al. [20], 2015	96.4	96.9	15.1	15.1	93.8	90.9	NR	NR	0	0 (0)	100
Kako et al. [21], 2018a	94.7	NR	21.4	NR	86.3	NR	NR	NR	0	0 (0)	100
Kako et al. [22], 2018b	95.7	95.7	17.8	17.6	93.9	88	NR	NR	0	0 (0)	100
Khor et al. [23], 2021	NR	NR	NR	NR	NR	NR	NR	NR	NR	1 (3)	100
Kocatepe et al. [24], 2021	95.6	96.6	25.7	24	95.1	93.9	125	120.2	NR	NR	NR
Long et al. [25], 2021	96	96	NR	NR	84	73	NR	NR	NR	0 (0)	100
Marchetti et al. [26], 2015	NR	NR	NR	NR	NR	NR	NR	NR	NR	0 (0)	100
O'Driscoll et al. [27], 2011	94.8	91.1	NR	NR	87.3	103.6	NR	NR	0	0 (0)	100
Smith et al. [28], 2022	NR	NR	NR	NR	NR	NR	NR	NR	0	0 (0)	100
Sutherl et al. [29], 2022	NR	NR	NR	NR	NR	NR	NR	NR	NR	0 (0)	100
Ting et al. [30], 2020	96.9	96.2	27.1	25.2	NR	NR	NR	NR	0	0 (0)	100

Eleven studies reported patient withdrawal rates of 0% [12,13,20-22,25-30], whereas three reported patient withdrawal rates ranging from 2.0 to 37% [18,19,23]. Thirteen studies had an adherence rate of 100% for fan therapy [12,13,18,20-23,25-30], one study had 48% [19], and one did not report adherence (Table 2) [24].

Discussion

To our knowledge, this is the first scoping review of fan therapy interventions and their safety and feasibility, especially in critically ill patients. We included 15 studies involving 505 participants and clarified that the available evidence for fan therapy is based primarily on patients with terminal-phase cancer, chronic-phase COPD, and cardiac disease. Based on the studies identified in our review, fan therapy appears to be safe when administered at rest and during exercise, with no adverse events reported. However, this scoping review found no evidence of fan therapy in critically ill patients.

We found that fan therapy mainly focused on the outcome of dyspnea relief in patients with terminal cancer, COPD, and cardiac disease. These interventions have three main characteristics. The first was a short-term intervention with a five-minute intervention period for dyspnea at rest, which was the most common intervention [12,13,18,20-22,28,30]. The second was a short-term intervention for dyspnea on exertion during exercise, such as a simple step test on an exercise step or ergometer [25-27]. This intervention was mainly performed in patients with respiratory diseases, such as COPD and ILD, and investigated improvement in exercise capacity as an outcome. Third, only three studies involved long-term interventions of 14 days or more using fan therapy [19,23,24].

Long-term interventions also have specific problems with reports of withdrawals due to changes in medical conditions [19]. The assessment tools used were the NRS [12,13,21,22,25,28], VAS [12,18,20], and mBorg [27,30] for short-duration interventions to evaluate safety in terms of changes in dyspnea intensity before and after a single intervention and physiological outcomes. By contrast, long-term interventions use dyspnea-12 to assess the quality of dyspnea in the physical and affective domains and secondary outcomes associated with improvement in dyspnea [23], such as health-related quality of life. Dyspnea assessment tools are broadly classified according to whether they evaluate the quantity or quality of dyspnea or its impact on life (quality of life-related). Those that can measure the expected outcome according to the purpose should be used [33-35]. For example, NRS, VAS, and respiratory distress observation scale (RDOS) should be used to evaluate the effect size of fan therapy. Likewise, to evaluate the quality of the effect, dyspnea-12 should be used. The most commonly used devices were handheld fans [12,18,19,23-25,28]. In long-term interventions, handheld fans are often used, and in the intervention plan, the patients are instructed to use them when their symptoms worsen. Long-term interventions require patients to come to terms with their illnesses. Handheld fans can be used indoors and outdoors. Indoors, they can be easily used to aid activities of daily living, such as washing and dressing, by relieving dyspnea during movement [36,37]. Standing fans [20-22] and table fans [13,29] are frequently selected in palliative care wards. We believe that the researchers selected a standalone fan because it becomes difficult for the patient to keep a handheld fan by themselves owing to the progression of their medical condition and other factors. Therefore, the device selected for use in fan therapy should be based on the patient’s medical condition and intended use.

Fan therapy has been suggested to relieve dyspnea through trigeminal activation of brain regions related to dyspnea, such as the insular cortex, anterior cingulate cortex, and amygdala, without improvement in breathing patterns or other physiological outcomes [38]. Based on the studies included in this review, no adverse events were reported, with no worsening of SpO_2_, respiratory rate, pulse rate, or blood pressure before or after fan therapy, suggesting that the use of fan therapy was safe. However, the results should be interpreted with caution, as only eight studies reported no adverse events. The possibility that some adverse events may have occurred in the unreported studies cannot be ruled out. Possible adverse events may include dry eyes and airways, although the likelihood and duration of these potential side effects are yet to be determined. Furthermore, it has been mentioned that fan therapy can alleviate dyspnea by “fooling the brain” and by “making” patients believe that their breathing is working better than it is, making it possible to miss dangerous signs in patients [39]. Therefore, in the future, rather than focusing only on the efficacy of fan therapy, the adverse events and physiological outcomes should be investigated to verify its safety.

The available evidence for fan therapy included studies on patients receiving oxygen therapy during the terminal phase, which confirmed its safety [20-23,26]. In the terminal phase, patients may be unable to maneuver themselves; thus, a standing fan or table fan could be chosen. Critically ill patients may also be unable to operate the fan independently due to sedation or ICU-acquired weakness. Therefore, even for critical care patients who cannot operate a fan on their own, fan therapy might be easily provided by selecting a standalone fan that can be operated by a healthcare professional when the patient requests to adjust the speed and direction of the fan. Electric fans are inexpensive and readily available, and handheld fans or table fans can be selected to minimize the space required for installation. Fan therapy does not require specialized knowledge to operate the fan and can be easily implemented by simply pressing the switch on the fan and blowing the wind onto the face. Currently, there are two ongoing studies recruiting participants admitted to the ICU [31,32]. However, further investigation into the safety of the intervention in this setting is warranted. It has been reported that patients with dyspnea have a slower weaning process and undergo prolonged ventilation [8]. Given this, the use of fan therapy for the treatment of dyspnea might improve post-intensive care syndrome (PICS) in patients by facilitating weaning and shortening the duration of ventilation.

Limitations

The present review has several strengths and limitations. The results are based on the evidence currently available from a comprehensive literature search. In addition, we employed a rigorous methodology followed by a written protocol developed a priori. The study limitations are as follows: the findings in this review are based on the analysis of a single intervention that was performed for a short duration (i.e., a few minutes). The studies included both chronic and terminal phase patients, but all the studies had small sample sizes. In addition, only two studies on long-term interventions differed in terms of duration and type of interventions, and there may be many unknown factors regarding safety and feasibility. Thus, the safety aspect of fan therapy cannot be generalized. However, this scoping review is also the first to identify a lack of evidence for fan therapy in critical care patients. In addition, this scoping review was not designed to assess methodological quality, and conclusions regarding the quality of the included studies should be interpreted with caution.

## Conclusions

In conclusion, previous studies on fan therapy have mainly focused on patients with terminal or chronic cancer and pulmonary and cardiac diseases. Fan therapy was performed at rest and during exercise using a handheld or standing fan, and changes in dyspnea and exercise capacity were investigated. In the studies included in our scoping review, there were no reports of worsening physiologic outcomes or adverse events. Although there are ongoing studies on using fan therapy for patients in a critical care setting, further studies are required to determine the safety of fan therapy in critically ill patients.

## Appendices

Appendix 1

**Table 3:** Preferred Reporting Items for Systematic reviews and Meta-Analyses extension for Scoping Reviews (PRISMA-ScR) Checklist JBI, Joanna Briggs Institute; PRISMA-ScR, Preferred Reporting Items for Systematic reviews and Meta-Analyses extension for Scoping Reviews. * Where sources of evidence (see second footnote) are compiled from, such as bibliographic databases, social media platforms, and Web sites. † A more inclusive/heterogeneous term used to account for the different types of evidence or data sources (e.g., quantitative and/or qualitative research, expert opinion, and policy documents) that may be eligible in a scoping review as opposed to only studies. This is not to be confused with information sources (see first footnote). ‡ The frameworks by Arksey and O’Malley (6) and Levac and colleagues (7) and the JBI guidance (4, 5) refer to the process of data extraction in a scoping review as data charting. § The process of systematically examining research evidence to assess its validity, results, and relevance before using it to inform a decision. This term is used for items 12 and 19 instead of "risk of bias" (which is more applicable to systematic reviews of interventions) to include and acknowledge the various sources of evidence that may be used in a scoping review (e.g., quantitative and/or qualitative research, expert opinion, and policy document).

SECTION	ITEM	PRISMA-ScR CHECKLIST ITEM	REPORTED ON PAGE #
TITLE			
Title	1	Identify the report as a scoping review.	p. 1
ABSTRACT			
Structured summary	2	Provide a structured summary that includes (as applicable): background, objectives, eligibility criteria, sources of evidence, charting methods, results, and conclusions that relate to the review questions and objectives.	p. 1
INTRODUCTION			
Rationale	3	Describe the rationale for the review in the context of what is already known. Explain why the review questions/objectives lend themselves to a scoping review approach.	p. 1
Objectives	4	Provide an explicit statement of the questions and objectives being addressed with reference to their key elements (e.g., population or participants, concepts, and context) or other relevant key elements used to conceptualize the review questions and/or objectives.	p. 1
METHODS			
Protocol and registration	5	Indicate whether a review protocol exists; state if and where it can be accessed (e.g., a Web address); and if available, provide registration information, including the registration number.	p. 1-2
Eligibility criteria	6	Specify characteristics of the sources of evidence used as eligibility criteria (e.g., years considered, language, and publication status), and provide a rationale.	p. 2
Information sources*	7	Describe all information sources in the search (e.g., databases with dates of coverage and contact with authors to identify additional sources), as well as the date the most recent search was executed.	p. 2
Search	8	Present the full electronic search strategy for at least 1 database, including any limits used, such that it could be repeated.	p. 2
Selection of sources of evidence†	9	State the process for selecting sources of evidence (i.e., screening and eligibility) included in the scoping review.	p. 2
Data charting process‡	10	Describe the methods of charting data from the included sources of evidence (e.g., calibrated forms or forms that have been tested by the team before their use, and whether data charting was done independently or in duplicate) and any processes for obtaining and confirming data from investigators.	Figure. 1
Data items	11	List and define all variables for which data were sought and any assumptions and simplifications made.	p. 2
Critical appraisal of individual sources of evidence§	12	If done, provide a rationale for conducting a critical appraisal of included sources of evidence; describe the methods used and how this information was used in any data synthesis (if appropriate).	-
Synthesis of results	13	Describe the methods of handling and summarizing the data that were charted.	p. 2
RESULTS			
Selection of sources of evidence	14	Give numbers of sources of evidence screened, assessed for eligibility, and included in the review, with reasons for exclusions at each stage, ideally using a flow diagram.	p. 2-3
Characteristics of sources of evidence	15	For each source of evidence, present characteristics for which data were charted and provide the citations.	p. 3
Critical appraisal within sources of evidence	16	If done, present data on critical appraisal of included sources of evidence (see item 12).	-
Results of individual sources of evidence	17	For each included source of evidence, present the relevant data that were charted that relate to the review questions and objectives.	p. 3
Synthesis of results	18	Summarize and/or present the charting results as they relate to the review questions and objectives.	p. 3-8
DISCUSSION			
Summary of evidence	19	Summarize the main results (including an overview of concepts, themes, and types of evidence available), link to the review questions and objectives, and consider the relevance to key groups.	p. 8
Limitations	20	Discuss the limitations of the scoping review process.	p. 9
Conclusions	21	Provide a general interpretation of the results with respect to the review questions and objectives, as well as potential implications and/or next steps.	p. 10
FUNDING			
Funding	22	Describe sources of funding for the included sources of evidence, as well as sources of funding for the scoping review. Describe the role of the funders of the scoping review.	p. 13

Appendix 2 

**Table 4:** Search strategy

Search	Search strategy
MEDLINE	#1 dyspnea[mh]
	#2 dyspnea[tiab]
	#3 dyspnoea[tiab]
	#4 breathless[tiab]
	#5 breathlessness[tiab]
	#6 labored breathing[tiab]
	#7 shortness of breath[tiab]
	#8 breath shortness[tiab]
	#9 inspiratory retraction[tiab]
	#10 breathing difficulty[tiab]
	#11 #3 NOT #2
	#12 #1 OR#2 OR #4 OR #5 OR #6 OR #7 OR #8 OR #9 OR #10 OR #11
	#13 air flow[tiab]
	#14 fan[tiab]
	#15 #13 OR #14
	#16 #12 AND #15
	((((((((((dyspnea[MeSH Terms]) OR (dyspnea[Title/Abstract])) OR (breathless[Title/Abstract])) OR (breathlessness[Title/Abstract])) OR (labored breathing[Title/Abstract])) OR (shortness of breath[Title/Abstract])) OR (breath shortness[Title/Abstract])) OR (inspiratory retraction[Title/Abstract])) OR (breathing difficulty[Title/Abstract])) OR ((dyspnoea[Title/Abstract]) NOT (dyspnea[Title/Abstract]))) AND ((air flow[Title/Abstract]) OR (fan[Title/Abstract]))
Embase	S1 EMB.EXACT.EXPLODE(“dyspnea”)
	S2 ab(dyspnea) OR ti(dyspnea)
	S3 ab(dyspnoea) OR ti(dyspnoea)
	S4 ab(breathless) OR ti(breathless)
	S5 ab(breathlessness) OR ti(breathlessness)
	S6 ab(labored breathing) OR ti(labored breathing)
	S7 ab(shortness of breath) OR ti(shortness of breath)
	S8 ab(breath shortness) OR ti(breath shortness)
	S9 ab(inspiratory retraction) OR ti(inspiratory retraction)
	S10 ab(breathing difficulty) OR ti(breathing difficulty)
	S11 S3 NOT S2
	S12 S1 OR S2 OR S4 OR S5 OR S6 OR S7 OR S8 OR S9 OR S10 OR S11
	S13 ab(fan) OR ti(fan)
	S14 ab(air flow) OR ti(air flow)
	S15 S13 OR S14
	S16 S12 AND S15
	((((((((((dyspnea/exp) OR (dyspnea:ti,ab)) OR (breathless:ti,ab)) OR (breathlessness:ti,ab)) OR ('labored breathing':ti,ab)) OR ('shortness of breath':ti,ab)) OR ('breath shortness':ti,ab)) OR ('inspiratory retraction':ti,ab)) OR ('breathing difficulty':ti,ab)) OR ((dyspnoea:ti,ab) NOT (dyspnea:ti,ab))) AND (('air flow':ti,ab) OR (fan:ti,ab))
CENTRAL	#1 MeSH descriptor: [dyspnea] explode all trees
	#2 (dyspnea):ti, ab, kw
	#3 (dyspnoea):ti, ab, kw
	#4 (breathless):ti,ab, kw
	#5 (breathlessness):ti, ab, kw
	#6 (labored breathing):ti, ab, kw
	#7 (shortness of breath):ti, ab, kw
	#8 (breath shortness):ti, ab, kw
	#9 (inspiratory retraction):ti, ab, kw
	#10 (breathing difficulty):ti, ab kw
	#11 #3 NOT #2
	#12 #1 OR #2 OR #4 OR #5 OR #6 OR #7 OR #8 OR #9 OR #10 OR #11
	#13 (air flow):ti, ab, kw
	#14 (fan):ti, ab, kw
	#15 #13 OR #14
	#16 #12 AND #15
	(((((((((([mh dyspnea]) OR (dyspnea:ti,ab)) OR (breathless:ti,ab)) OR (breathlessness:ti,ab)) OR ("labored breathing":ti,ab)) OR ("shortness of breath":ti,ab)) OR ("breath shortness":ti,ab)) OR ("inspiratory retraction":ti,ab)) OR ("breathing difficulty":ti,ab)) OR ((dyspnoea:ti,ab) NOT (dyspnea:ti,ab))) AND (("air flow":ti,ab) OR (fan:ti,ab))
CINAHL	S1 MH (dyspnea)
	S2 TI (dyspnea) OR AB (dyspnea)
	S3 TI (dyspnoea) OR AB (dyspnoea)
	S4 TI (breathless) OR AB (breathless)
	S5 TI (breathlessness) OR AB (breathlessness)
	S6 TI (labored breathing) OR AB (labored breathing)
	S7 TI (shortness of breath) OR AB (shortness of breath)
	S8 TI (breath shortness) OR AB (breath shortness)
	S9 TI (inspiratory retraction) OR AB (inspiratory retraction)
	S10 TI (breathing difficulty) OR AB (breathing difficulty)
	S11 S3 NOT S2
	S12 S1 OR S2 OR S4 OR S5 OR S6 OR S7 OR S8 OR S9 OR S10 OR S11
	S13 TI (air flow) OR AB (air flow)
	S14 TI (fan) OR AB (fan)
	S15 S13 OR S14
	S16 S12 AND S15
	(((((((((((MH dyspnea+)) OR ((TI dyspnea OR AB dyspnea))) OR ((TI breathless OR AB breathless))) OR ((TI breathlessness OR AB breathlessness))) OR ((TI "labored breathing" OR AB "labored breathing"))) OR ((TI "shortness of breath" OR AB "shortness of breath"))) OR ((TI "breath shortness" OR AB "breath shortness"))) OR ((TI "inspiratory retraction" OR AB "inspiratory retraction"))) OR ((TI "breathing difficulty" OR AB "breathing difficulty"))) OR (((TI dyspnoea OR AB dyspnoea)) NOT ((TI dyspnea OR AB dyspnea)))) AND (((TI "air flow" OR AB "air flow")) OR ((TI fan OR AB fan)))
WHO-ICTRP	Condition: dyspnea OR dyspnoea OR breathless OR breathlessness OR labored breathing OR shortness of breath OR breath shortness OR inspiratory retraction OR breathing difficulty
	Intervention: fan OR air
ClinicalTrials.gov	Condition or disease: Dyspnea OR dyspnoea OR breathless OR breathlessness OR labored breathing OR shortness of breath OR breath shortness OR inspiratory retraction OR breathing difficulty
	Intervention: Fan OR Air flow

**Table 5:** Characteristics of studies excluded from qualitative and quantitative synthesis

Study	Reason for exclusion
Baltzan et al., Am J Respi Crit Care Med. 2000;161:A59	Congress abstract
Cassidy et al., Ir J Med Sci. 2017;186:S387–S445	
Johnson et al., Am J Respi Crit Care Med. 2017;195	
Long et al., Thorax 2018;73:A252	
Smith et al., Euro Respi J. 2016;48:719	
Smith et al., Respirology. 2017;22:147	
Derry et al., Euro Respi J. 2006;28:71s[E504]	Wrong intervention
Puspawati et al., Asia Pac J Oncol Nurs. 2017;4(2):162-167	
Swan et al., J Pain Symptom Manage. 2019;57(6):1051-1061	
Wong et al., Hong Kong Physiotherapy J. 2013;31(2):101	
Derry et al., ISRCTN94278636	Protocols without results
Yan et al., ChiCTR-INR-16009453	
Swan et al., ISRCTN12024425	
Khor et al., ACTRN12618001949279	
Lehto et al., NCT05257850	
Pannu et al., NCT05416437	
Nagumo et al., UMIN000039821	
Kanezaki et al., ERJ Open Res. 2019;15;5(4)	Wrong population
Schwartzstein et al., Am Rev Respir Dis. 1987;136(1):58-61	
